# Association of Blood Pressure Mean and Variability with Hippocampal Subfield Volumes in Cerebral Amyloid Angiopathy with Mild Cognitive Symptoms

**DOI:** 10.1002/alz.093298

**Published:** 2025-01-09

**Authors:** Lukas SVEIKATA, Maria Clara Zanon Zotin, Dorothee Schoemaker, Valentina Perosa, Frederic Assal, Steven M. Greenberg, Anand Viswanathan

**Affiliations:** ^1^ Geneva University Hospitals, Geneva Switzerland; ^2^ Massachusetts General Hospital, Harvard Medical School, Boston, MA USA; ^3^ Massachusetts General Hospital, Boston, MA USA

## Abstract

**Background:**

The interaction between Alzheimer's pathology and cerebral amyloid angiopathy (CAA), a common cerebral small vessel disease, plays a crucial role in cognitive impairment development in older adults. Recent studies suggest that increased blood pressure (BP) variability may affect subclinical vascular disease and cognitive decline. However, it is currently unknown whether a particular BP profile is linked to hippocampal subfield volumes, which are a significant feature of cognitive decline in neurodegenerative diseases. Therefore, we conducted a study to investigate the relationship between BP profile and hippocampal subfields in nondemented subjects with CAA.

**Method:**

We investigated the relation between BP mean and variability and hippocampal neurodegeneration in a memory clinic cohort of non‐demented participants with possible and probable cerebral amyloid angiopathy (CAA), diagnosed using the modified Boston criteria v1.5. Linear regression models were used to assess the associations between BP profile and hippocampal volumes. The subfield volumes were measured using an automated method on T‐1 weighted sequence using FreeSurfer v6.0. The BP profile was defined using serial outpatient BP measurements five years prior to 3T research MRI. The BP variability was calculated using the coefficient of variation (standard deviation/mean).

**Result:**

We have included 59 participants (74.3 ± 6.8 y, 9/50 F/M) who underwent a median of 12 (IQR 26) outpatient BP measurements five years prior to index MRI. We found a significant association between mean BP and all measured hippocampal subfields, notably the CA1: b=‐032, p=0.008, CA3: b = ‐0.24, p=0.049, CA4: b=‐0.34, p=0.003, and the subiculum: b=‐0.29, p=0.006), adjusted for age and brain volume normalized for total intracranial volume. However, we found no significant association with long‐term BP variability

**Conclusion:**

We found significant associations between mean BP and hippocampal subfields volumes. Our results suggest that elevated BP, but not its variability, is a modifiable risk factor for preventing hippocampal degeneration in the context of CAA‐related cognitive impairment. Further pre‐planned analyses will evaluate the associationa in a subgroup of participants with mild dementia and non‐CAA participants.

